# Organ-preserving endoscopic resection of a large colorectal lesion causing McKittrick–Wheelock syndrome

**DOI:** 10.1055/a-2590-8284

**Published:** 2025-05-22

**Authors:** Shi Jie Looi, Omer Ahmad, Edward Seward, Roser Vega

**Affiliations:** 1 8964 Gastroenterology Department, University College London Hospitals NHS Foundation Trust, London, United Kingdom

McKittrick–Wheelock syndrome is a rare but life-threatening condition characterised by severe diarrhoea, electrolyte disturbances, and kidney injury caused by colorectal tumours 1 . The majority of reported cases have been managed by surgical resection 2 . We demonstrate a case of McKittrick–Wheelock syndrome managed endoscopically by speedboat-assisted endoscopic submucosal dissection (S-ESD). S-ESD involves the use of a novel endoscopic electrosurgical device combining advanced bipolar radiofrequency for dissection and microwave energy for coagulation ( Video 1 ). This technique was selected to enable en-bloc resection with the potential for organ preservation, while minimising the risks associated with surgery below the peritoneal reflection, particularly in an elderly patient with multiple co-morbidities.

Speedboat-assisted endoscopic submucosal dissection (S-ESD) of a large colorectal lesion causing McKittrick–Wheelock syndrome.Video 1

The lesion was removed en-bloc completely by S-ESD ( Fig. 1 ). Our patient had an uneventful recovery without any immediate or delayed complications. Histology confirmed R0 resection of a tubulovillous adenoma with low-grade dysplasia and focal high-grade dysplasia. S-ESD using Speedboat is a safe alternative to surgery for the management of McKittrick–Wheelock syndrome especially with lesions below the peritoneal reflection to minimise complications associated with surgery.

**Fig. 1:**
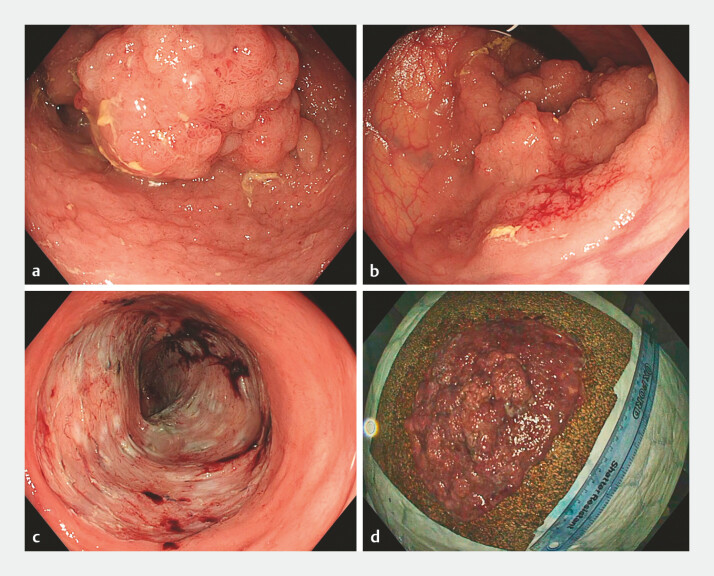
Endoscopic resection of a large colorectal lesion causing McKittrick–Wheelock syndrome. **a, b** , Laterally spreading tumour extending from the rectum beyond the rectosigmoid junction. **c** Resection bed following Speedboat-assisted endoscopic submucosal dissection. **d** Final specimen measuring over 17 cm.

Endoscopy_UCTN_Code_TTT_1AQ_2AD_3AD

## Notes

Endoscopy E-Videos https://eref.thieme.de/e-videos
*E-Videos* is an open access online section of the journal *Endoscopy* , reporting on interesting cases and new techniques in gastroenterological endoscopy. All papers include a high-quality video and are published with a Creative Commons CC-BY license. Endoscopy E-Videos qualify for HINARI discounts and waivers and eligibility is automatically checked during the submission process. We grant 100% waivers to articles whose corresponding authors are based in Group A countries and 50% waivers to those who are based in Group B countries as classified by Research4Life (see: https://www.research4life.org/access/eligibility/ ). This section has its own submission website at https://mc.manuscriptcentral.com/e-videos .
